# Supplementary material for: Characterization and Food Application of the Novel Lytic Phage BECP10: Specifically Recognizes the O-polysaccharide of Escherichia coli O157:H7
Source: Viruses. 2021 Jul 27;13(8):1469. doi: 10.3390/v13081469 (PMC8402813; doi:10.3390/v13081469)
Supplement: Supplementary file 1 [file viruses-13-01469-s001.zip › viruses-1283536-supplementary Figure S1.pdf]

Characterization and Food Application of the Novel Lytic Phage BECP10: Specifically Recognizes the O-polysaccharide of *Escherichia coli* O157:H7

Do-Won Park · Jong-Hyun Park*

Department of Food Science and Biotechnology, Gachon University, Seongnam 13120, Korea; sgm01006@naver.com

Contact Information for Corresponding Author:

Direct inquiries to author Jong-Hyun Park

Department of Food Science and Biotechnology, Gachon University, Seongnam 13120, Korea.

Tel.: +82-31-750-5523, Fax.: +82-31-750-5273, E-mail: p5062@gachon.ac.kr

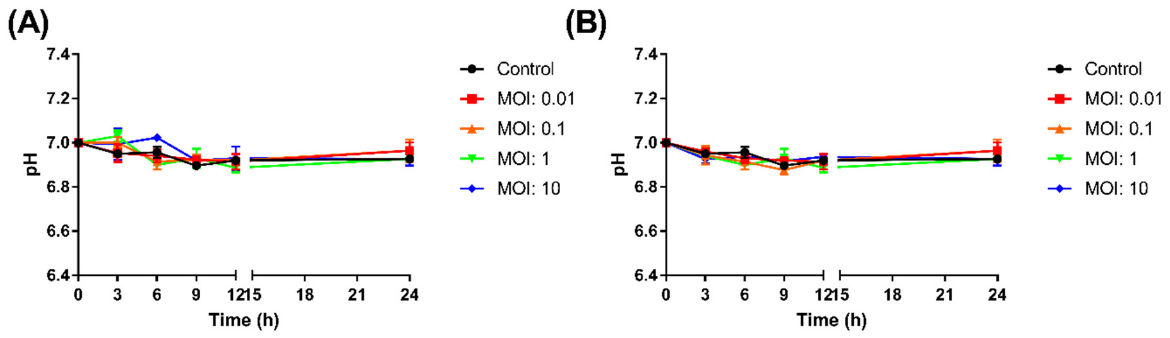

Supplementary Figure S1. Changes of growth medium pH during bacterial challenge assay. (A) pH changes after inoculation with phage ECP26 according to various MOIs at 37 °C. (B) pH changes after inoculation with phage BECP10 according to various MOIs at 37 °C. Phages were inoculated at 3 h after bacterial growth and the pH was monitored every 3 h for 24 h. SM buffer was used as the negative control.
